# Could Ergothioneine Aid in the Treatment of Coronavirus Patients?

**DOI:** 10.3390/antiox9070595

**Published:** 2020-07-07

**Authors:** Irwin K. Cheah, Barry Halliwell

**Affiliations:** 1 Department of Biochemistry, Yong Loo Lin School of Medicine, National University of Singapore, Singapore 117596, Singapore; bchickm@nus.edu.sg; 2 Life Science Institute, Neurobiology Programme, Centre for Life Sciences, National University of Singapore, Singapore 117456, Singapore

**Keywords:** ergothioneine, COVID-19, antioxidant, coronavirus, SARS, inflammation, cytokine, NETs

## Abstract

Infection with SARS-CoV-2 causes the coronavirus infectious disease 2019 (COVID-19), a pandemic that has, at present, infected more than 11 million people globally. Some COVID-19 patients develop a severe and critical illness, spurred on by excessive inflammation that can lead to respiratory or multiorgan failure. Numerous studies have established the unique array of cytoprotective properties of the dietary amino acid ergothioneine. Based on studies in a range of in vitro and in vivo models, ergothioneine has exhibited the ability to modulate inflammation, scavenge free radicals, protect against acute respiratory distress syndrome, prevent endothelial dysfunction, protect against ischemia and reperfusion injury, protect against neuronal damage, counteract iron dysregulation, hinder lung and liver fibrosis, and mitigate damage to the lungs, kidneys, liver, gastrointestinal tract, and testis, amongst many others. When compiled, this evidence suggests that ergothioneine has a potential application in the treatment of the underlying pathology of COVID-19. We propose that ergothioneine could be used as a therapeutic to reduce the severity and mortality of COVID-19, especially in the elderly and those with underlying health conditions. This review presents evidence to support that proposal.

## 1. Introduction: Coronaviruses and COVID-19

Coronaviruses consisting of an enveloped, nonsegmented positive-sense RNA are the largest group of viruses [1]. They cause a range of respiratory and intestinal infections in animals and humans but were not considered to be highly pathogenic in humans until two novel variants, the severe acute respiratory syndrome coronavirus (SARS-CoV) in 2002 [2], and a decade later, the Middle East respiratory syndrome (MERS) CoV [3], brought global awareness to their infectivity and lethality. In December 2019, a novel coronavirus with high genetic similarity (~79%) to the original SARS CoV [4] emerged from Wuhan, China, and almost brought the world to a standstill with its high infectivity. The infection of the human respiratory epithelium by SARS CoV-2 results in the coronavirus infectious disease 2019 (COVID-19). At the present time of writing, there are more than 11 million diagnosed cases and close to 530,000 deaths globally (WHO, COVID-19 situation reports). However, the numbers are escalating daily. Present information on the transmission of COVID-19 suggests this is primarily through direct and indirect (contaminated surfaces or objects [5]) contact and short-range (within 6 feet) droplet spray transmission when an infected individual speaks, coughs, or sneezes at close proximity (US-CDC). There is also the debatable issue of longer-range aerosolized transmission [5,6] and also transmission through the fecal/oral route, which has yet to be proven [7].Similar to the SARS CoV, the spike (S) glycoproteins found on the envelope of SARS-CoV-2 bind to human angiotensin-converting enzyme 2 (ACE2) receptors found in the nasal epithelium and elsewhere in the body [8]. The spike protein comprises subunits S1 and S2, facilitating receptor binding and membrane fusion, respectively, and requires cleavage at two sites for cellular translocation by host proteases transmembrane serine protease-2 (TMPRSS2) [9] and the proprotein convertase furin [10,11]. Studies into the expression of ACE2 receptor and TMPRSS2/furin reveal that both are present in a wide range of tissues, including the airways, cornea, esophagus, ileum, colon, liver, gallbladder, heart, kidneys, and testis [12]. However, nasal swabs indicate a higher viral load than throat swabs, indicating that nasal epithelium is the primary site of infection [13]. Despite the high genetic similarity between SARS-CoV and SARS-CoV-2, sera from recovered COVID-19 patients have demonstrated limited cross-neutralization with SARS-CoV and vice-versa [8,14], indicating that prior immunity against one virus will likely not provide immunity against the other.The clinical severity of symptoms due to COVID-19 varies considerably, with most patients ranging from asymptomatic to mild or moderate illness. However, a minority of infected individuals (especially elderly or those with underlying chronic disorders) progress to severe and critical illness. The most common clinical symptoms for COVID-19 are similar to earlier coronavirus infections (i.e., the SARS CoV and MERS CoV), with fever present in close to 90% of cases and more than two-thirds developing cough [15]. Some also present a range of symptoms including, but not restricted to, shortness of breath (~19%; dyspnea), sore throat (~14%; pharyngitis), loss of olfactory (anosmia) and gustatory (ageusia) functions, muscle pain (myalgia), nausea, diarrhea, vomiting, and neurological complications [15,16,17]. The loss of olfactory and gustatory functions has been reported in approximately 34% of cases [17] and is often an early event prior to the presentation of other symptoms [18].

Based on studies of close to 45,000 patients in China, estimates show that around 81% of cases are asymptomatic or display mild symptoms, while about 14% develop more severe symptoms, and 5% fall critically ill [19]. The estimated mortality rate for COVID-19 is around 3.4%, which is far lower than the earlier MERS CoV (34.4%) or SARS CoV (11%). However, fatality rates vary quite considerably between countries, ranging from less than 1% to greater than 7% [20], which are related to the implementation of mass screenings in some areas [21], the capacity of healthcare systems, and the proportion of elderly in the population, which is especially pertinent to Italy, with ~23% of the population over the age of 65 [20].

A strong correlation of mortality with increasing age has been shown, with around 17.5% (% of total cases for the age) over the age of 80 succumbing to the disease, whilst the mortality is ~10.5% for those aged between 70–79, ~3.5% for those aged between 60–69, and only around 0.5% for individuals under 60 years of age based on the average of case-fatality rates from Italy and China [20]. Greater than 80% of deaths due to COVID-19 occur in individuals over the age of 60 [22]. The most common cause of mortality is believed to be acute respiratory distress syndrome (ARDS), often leading to other complications such as organ failure or exacerbation of comorbidities, which likely explains the correlation with age since those with advanced age and pre-existing comorbidities have a higher risk of developing ARDS [23]. However, much remains unknown about the actual underlying pathophysiology of the disease, and more data are urgently needed to further understand the processes leading to morbidity and mortality in COVID-19.

## 2. Biology of Ergothioneine

Ergothioneine (ET; refer to [24,25,26] for detailed overviews of ET) is a naturally occurring dietary amino acid that is able to accumulate in most cells and tissues in the body due to the presence of the organic cation transporter novel-type 1 (OCTN1), which has a specific role in ET transport as first uncovered by Grundemann et al. [24,26,27,28]. Whilst ET biosynthesis has only been demonstrated to date in some fungi and bacteria, it is ubiquitously found in animals, and it is avidly absorbed following oral consumption and accumulated in our bodies, suggestive of an important physiological role [29,30,31,32]. ET is not rapidly metabolized or excreted in urine, and the same transporter, that is, OCTN1, is responsible for its distribution around the body [24,28,29]. The long-half life and ability to accumulate in the body are partially due to the fact that ET predominantly exists in the thione tautomer at physiological pH, giving it greater stability compared to other thiols such as glutathione [25]. ET has a wide range of unique properties, conferring a variety of cytoprotective capabilities which, amongst others, includes the ability to scavenge reactive oxygen and reactive nitrogen species (ROS/RNS) such as hydroxyl radicals (^•^OH) [33], hypochlorous acid (HOCl) [33], singlet oxygen (^1^O_2_) [34,35], and peroxynitrite (ONOO^−^) [36]. However, a wide range of other studies have demonstrated that ET can, amongst other things, modulate inflammation [37,38,39], chelate divalent metal cations such as iron and copper (thereby decreasing the ability of these metal ions to stimulate oxidative damage) [33,40,41,42], protect against UV radiation-induced damage [43,44], inhibit expression of vascular adhesion proteins [45,46], inhibit myeloperoxidase activity [47], protect against the phagocyte respiratory burst [48,49], induce the expression of heat shock protein-70 [39,50], promote neuronal differentiation [51,52,53], and prevent lung [54] and liver [55,56] fibrosis. These features of ET and their application against various pathological aspects of COVID-19 will be discussed in greater detail throughout this review to demonstrate the multifunctional capabilities of ET as a potential therapeutic for coronavirus infection. Moreover, studies have suggested that ET is preferentially accumulated in tissues predisposed to oxidative stress and inflammation and may even be concentrated at sites of tissue injury by cellular modulation of the levels of OCTN1 [24,32,56,57]. These unique capabilities of ET, which have been reported in a wide range of studies in cells, animal models, and also population studies, suggest that the cytoprotective abilities of ET may be helpful against disorders such as dementia [58,59,60], depression [61], atherosclerosis [45,46], cardiovascular disorders [46,62,63,64], nonalcoholic fatty liver disease [56], preeclampsia [65,66], ischemia-reperfusion injury [39,50,67], and, especially relevant in this case, ARDS [68]. This list is by no means exhaustive, with new applications for ET constantly being identified even in other areas of human health, such as improving the efficacy of cancer vaccines [69] and protection against chemotherapy-induced peripheral neuropathy [70]. ET has even been suggested to be a longevity nutrient that can reduce the risk of frailty in the elderly [60,71,72,73].

Indeed, low blood plasma concentrations of ET are associated with age-related disorders [60,64,74], suggesting that ET may be beneficial as a therapeutic or prophylactic supplement to reduce the risk of these disorders. Furthermore, we and others have identified that blood ET levels decline with advancing age, possibly predisposing elderly individuals to an increased risk of age-related disorders [60,72,75], which may include frailty, cardiovascular disorders, and neurodegenerative disease.

## 3. How Might Ergothioneine Be Beneficial in Alleviating COVID-19?

### 3.1. Use of Antioxidants to Reduce COVID-19 Severity

During the initial stages of an invasion by SARS-CoV-2 (and other pathogens), the immune response plays a vital role in eradicating the virus, a process in which oxygen radicals and other ROS play a key role. In particular, neutrophils can deactivate the invading pathogen through a respiratory burst, which involves the generation of ROS, such as superoxide (O_2_^•−^), H_2_O_2_, and HOCl (reviewed in [76]). However, in many patients, the excessive activation of the immune response contributes to damage and many of the symptoms experienced. Indeed, excessive damage by oxygen radicals and other ROS, such as HOCl and ONOO^−^ and inflammatory mediators, is known to be a key player in many chronic inflammatory diseases and the progression of infections such as influenza, dengue, and other coronaviruses [76,77,78,79,80,81]. While the generation of ROS is an essential component of the human immune response, their excessive production can damage biological molecules (i.e., oxidative damage), and this process contributes to tissue damage and possibly further propagation of a vicious cycle of oxidative stress [76].

Since ROS can be key mediators of damage and disease progression [76], antioxidants have been suggested as a means of stemming disease duration and severity of COVID-19. An early report from the Shanghai Medical Association endorsed the use of high doses of ascorbic acid in patients hospitalized with COVID-19 to reduce the severity of symptoms. Some studies have shown that during severe infections, the levels of vitamin C become depleted [82], and patients supplemented with vitamin C fared better than placebo controls [83] and spent less time on mechanical ventilation and in the ICU [84]. As such, numerous clinical trials in China, Italy, and the US have commenced investigating the use of intravenously administered high doses (3–12 g/day) of vitamin C (clinicaltrials.gov study: NCT04264533/NCT04323514). These doses are more than 133 times the recommended daily intake for an adult by the European Food and Safety Authority [85]. When given orally, the plasma ascorbate levels plateau when given more than 100 mg/day [85] through renal clearance, suggesting that there may be little justification for the oral administration of high dose ascorbic acid [86]. Moreover, due to the rapid clearance of IV-administered ascorbic acid at high dose, with a half-life of about 2 h, the IV administration would need to be repeated multiple times a day to maintain the desired high concentrations in plasma [87]. This is due to glomerular filtration and excretion with insignificant reuptake [88]. Although ascorbic acid is considered a powerful antioxidant, caution should be taken in its application as it may also facilitate pro-oxidative reactions with iron or copper [76,89], especially during disorders that may have dysregulation of iron metabolism, such as may be found in ARDS [90]. Indeed, there is evidence that IV ascorbate exerts pro-oxidant actions in vivo [91,92,93], while the evidence that ascorbate is an important antioxidant in vivo in the body is quite weak, except perhaps in the respiratory tract, as relevant here [76,94,95].

By contrast, ET is actively absorbed and accumulated at high concentrations in the body following oral consumption, with high retention due to renal reabsorption [29]. Moreover, our studies in animals show that ET is distributed and accumulated in most (if not all) organs and tissues of the body, with preferential accumulation in tissues predisposed to high levels of oxidative stress [32]. As mentioned earlier, numerous studies have shown the ability of ET to scavenge ROS/RNS, such as ^•^OH, HOCl, ONOO^−^ and ^1^O_2_, at very high rates [33,36,96,97], and it was also claimed to protect biomolecules against damage from O_2_^•−^ [43,98,99] and H_2_O_2_ [97,100]. Indeed, a wide range of in vitro and in vivo studies have shown that ET can protect biomolecules from oxidative damage and demonstrate cytoprotection through antioxidants and other capabilities (that will be explored later) when systems are exposed to a range of stressors [99,100]. Conversely, silencing the gene encoding OCTN1 predisposes cells and animals to higher levels of oxidative damage [101,102,103]. This suggests that elevating tissue levels of ET in the body will be beneficial in protecting from oxidative damage [57,104]. Moreover, ET has a high bioavailability, being actively taken up into cells and tissues where it is needed. Furthermore, some evidence suggests a feedback mechanism to modulate the expression of OCTN1, which will further direct ET to sites of tissue injury [57,105]. This, combined with the demonstrated clinical safety of ET (Section 3), presents a strong case for the clinical application of ET to limit mortality and tissue damage in severely ill COVID-19 patients.

### 3.2. Direct Inhibition of Viral Replication

To date, no studies have looked at the potential of ET to directly inhibit coronavirus replication. A recent review by Suwannarach et al. [106] highlights the potential for fungal extracts, of which a major active component is ET, to demonstrate inhibitory activity on other RNA viruses such as HIV-1 and suggests a possible application for the inhibition of binding or replication of coronaviruses. Likewise, Xiao et al. [107] demonstrated that pure ET was able to dose-dependently inhibit the pro-fs (human immunodeficiency virus-1 protein; HIV-1) activation of the HIV long terminal repeat region (which serves as a promoter‒enhancer for viral genome integration in host DNA) using an in vitro assay, suggesting that ET directly inhibits HIV transcription. Recently, another study demonstrated that water-extracts of a common edible mushroom, of which a major active component is ET, inhibited NS3/4A protease activity (~90% inhibition) and hepatitis C viral (HCV) genome replication (~93% decrease in viral load) [108]. Likewise, another study using *Agaricus blazei* mushroom extracts slightly decreased peripheral blood viral load in hepatitis C patients [109]. These studies are a small representation of the literature on the abilities of fungal extracts to inhibit viral proteases, preventing viral binding and replication. Of course, these studies do not prove that ET is the component responsible (although mushrooms are the primary dietary source of ET in the body), and further studies are needed to evaluate if ET could be beneficial in directly reducing SARS-CoV-2 uptake or replication. This section highlights the urgent need for studies to evaluate the inhibitory activity of ET on SARS-CoV-2 uptake and replication and its direct antiviral activity.

### 3.3. Modulating Inflammation, Cytokine Storm, and Acute Respiratory Distress Syndrome

The inflammatory response is a key mediator of viral clearance and recovery through neutralizing antibodies, neutrophils, macrophages and T-cells. During the initial infection, if the immune system is not able to hold back the virus at the upper respiratory system, the virus makes its way to the lungs, whereby the severity of infection may significantly increase. At the lungs, viral infiltration of cells and subsequent cell death (often by pyroptosis—a programmed cell death initiating inflammatory responses) triggers a pulmonary immune response, drawing macrophages and monocytes to the site of damage [110]. Meanwhile, cytokine release leads to priming of the adaptive T- and B-cell immune response. In a healthy individual, this basic immune response can typically halt the viral infection before the virus is able to greatly replicate and spread, preventing an overzealous immune response and limiting tissue damage. However, in certain patients, a dysfunction of the immune response, possibly due to underlying conditions, can lead to poor suppression of the virus and an excessive inflammatory response. Indeed, marked elevations of serum levels of inflammatory cytokines are seen in COVID-19 patients, including IL-1β, IL-2, IL-7, IL-8, IL-9, IL-10, IL-17, IFNγ, IP-10, TNF-α, and MCP-1 [111].

The widespread excessive inflammation (termed cytokine storm) in the lungs can cause damage (in part via excessive ROS production and protease secretion) to the alveoli and leads to pulmonary edema (fluid build-up in the lungs), limiting gas exchange [112]. This leads to respiratory distress (dyspnea), and in ~10–15% cases, progresses further to ARDS, which is often fatal [23,113]. Studies show that ARDS leading to respiratory failure is the leading cause of death in COVID-19 patients, with estimates ranging between 70–90% [114,115,116]. However, reports indicate that all patients who have died due to COVID-19 had some form of respiratory damage. Furthermore, the massive release of proinflammatory cytokines (in particular, TNF-α, IL-1β, and IL-6) from lungs and other tissues triggers a systemic cytokine storm that may lead to sepsis and multiorgan failure [117,118].

Some studies have demonstrated the anti-inflammatory properties of ET and its potential to modulate proinflammatory cytokines (e.g., by inhibiting palmitic acid-induced IL-6 expression and preventing muscle cell death in vitro [37] and the significant reduction of serum IL-1β and TNF-α levels due to lung and intestinal ischemia-reperfusion injury) in mice relative to untreated controls [39,67].

A key study employing a rat model of ARDS (cytokine insufflation, which recapitulates many of the pathological features of ARDS and is commonly used as a model to investigate potential treatments), revealed that both pre- (15 and 150 mg/kg) and post- (150 mg/kg) treatment with ET was protective, and led to decreases in lung injury and inflammation (lung neutrophils) relative to untreated controls [68]. The authors cite the free radical scavenging (as detailed earlier), divalent metal chelating, and anti-inflammatory properties of ET as the main mechanisms of protection against ARDS [68]. In particular, for the latter, is the inhibition of NF-κB activation and IL-8 expression, which are involved in macrophage and neutrophil recruitment to the lungs. Pretreatment of lung alveolar epithelial cells with ET was able to prevent both H_2_O_2_ and TNF-α-induced activation of NF-κB and downregulation of IL-8 expression [38]. Studies have also verified the ability of ET to inhibit NF-κB expression in rat pheochromocytoma (PC-12) cells [119].

A key component in tissue injury due to excessive inflammation is the enzyme myeloperoxidase (MPO), a major component of neutrophils [76], which catalyzes the production of reactive molecules, such as HOCl from H_2_O_2_, to help destroy invading pathogens. Higher neutrophil counts in the blood of COVID-19 patients have been linked to higher severity and likelihood of mortality and may even be used as an early means of predicting severe outcomes in patients [16,120]. Neutrophils also produce neutrophil extracellular traps (NETs; a mesh of DNA fibers, histones, and antimicrobial proteins), which, in addition to promoting further inflammation, are known to contribute to microvascular thrombosis, sepsis, and multiorgan failure [121,122]. While NETs are important in the removal of pathogens during infection, they have also been shown to play a role in diseases such as preeclampsia [123] and transfusion-related acute lung injury [124]. Not only are MPO and ROS required for NET formation, but MPO is actually a component of the NET, generating further ROS in the propagation of a vicious cycle [125]. Elevated serum NETs have been seen in COVID-19 patients (as indicated by elevations in cell-free DNA, MPO-DNA, and citrullinated histone H3 biomarkers). Furthermore, sera from COVID-19 patients stimulated NETosis (activation and release of NETs) when added to normal neutrophils [120]. NETosis is suggested to be a key player in promoting severe outcomes in COVID-19, including the cytokine storm, ARDS, thrombus-associated events, and excessive tissue damage due to inflammation and ROS [113].

ET has been suggested to inhibit MPO activity even at concentrations of 1 µM. In addition, it is well known to be able to scavenge MPO-derived reactive halogenating molecules such as HOCl [33] at higher rates than ascorbic acid and glutathione [47]. It was also further demonstrated that ET protected DNA from halogenation in vitro and in vivo (using a UV-B-induced mouse skin model for neutrophil accumulation) and decreased associated histopathological changes to skin [47]. Servillo et al. [48] also demonstrated the ability of ET to scavenge HOCl during the respiratory burst using ex vivo human neutrophils activated with phorbol-12-myristate-13-acetate. These findings suggest that ET might interfere with the propagation of neutrophil-driven NETosis.

Collectively, the evidence here highlights the potential for ET administration to reduce the severity of lung inflammation through the inhibition of pro-inflammatory cytokines, MPO activity, and NETosis, which thereby protects against ARDS and helps prevent a cytokine storm during COVID-19. This is especially applicable to patients predicted to have more severe outcomes, high levels of neutrophils, or those with underlying comorbidities.

### 3.4. Protection against Endothelial Dysfunction

A recent report [126] suggests that viral damage to the endothelial cells lining blood vessels may lead to sepsis and multiple organ failure. Indeed, vascular endothelial cells express ACE2 receptors, facilitating the uptake of the virus [12]. This can lead to immune-mediated cell death and endothelial dysfunction [126]. Dysfunction of the endothelial system is central to a wide range of cardiovascular diseases, including hypertension, coronary artery disease, chronic heart failure, peripheral vascular disease, diabetes, chronic kidney failure, and is also central to the pathology of viral infections [127]. Indeed, dysfunction of the endothelium leads to the promotion of vasoconstriction, proinflammatory cytokines, and prothrombic state, contributing to the hypercoagulative state discussed in the next section [127]. The immune-mediated process by which SARS-CoV-2 infiltrates and damages the vascular endothelium is similar to that of the epithelial cells (pneumocytes) lining the alveoli, causing lung injury, dyspnea, and in severe cases, ARDS [128]. The virus can penetrate the endothelium of several organs, as indicated by the presence of viral bodies, leading to endotheliitis in various organs (lung, heart, kidneys, liver, and intestines) [126]. Studies have also shown that the endothelium is central to the promotion of a cytokine storm during viral infections by orchestrating immune cell infiltration and cytokine production [129]. Of additional concern is that sustained injury to the vascular endothelium may lead to an elevated risk of cardiovascular events, such as hypertension, atherosclerosis, stroke, or myocardial infarction.

Earlier studies have demonstrated that ET can be taken up by human endothelial cells and protects against oxidative stress and inflammation caused by a range of stressors [45,98,130]. Furthermore, studies have identified that higher blood ET levels are associated with a lower risk of cardiometabolic diseases and mortality [64]. Our pharmacokinetic studies in healthy volunteers have demonstrated that oral administration of ET at 25 mg/day for 1 week can raise blood plasma levels by up to 3-fold [29]. A study by Li et al. [130] demonstrated that human brain microvascular endothelial cells (HBMEC) are capable of taking up ET. Using isolated rat basilar arteries and HBMEC, they demonstrated that ET supplementation was able to prevent pyrogallol, hypoxanthine/xanthine oxidase, or high glucose inhibition of acetylcholine-induced relaxation and oxidative stress-induced HBMEC death, respectively. Similarly, in isolated rat thoracic aortas, ET significantly reduced superoxide generation by hypoxanthine/xanthine oxidase or the superoxide dismutase-1 inhibitor diethyldithiocarbamate and demonstrated a concentration-dependent relaxation of aortic rings [63]. These protective effects were negated by removing the endothelium [63] or silencing the ET transporter (OCTN1) using siRNA [130]. Other studies have also recognized the cytoprotective capabilities of ET through the prevention of endothelial cell death due to the administration of hydrogen peroxide, paraquat, high glucose, or oxidized-LDL [45,62,98]. ET administration increased the expression of glutathione reductase, catalase, and superoxide dismutase 1 and 2, and decreased NADPH oxidase 1 expression in endothelial cells [130].

Incubation of endothelial cells with the proinflammatory cytokine IL-1β or oxidized-LDL induces inflammatory cell adhesion molecules (CAMs). However, preloading human aortic or umbilical vein endothelial cells (HAECs/HUVECs) with ET significantly reduced the expression of vascular (VCAM-1), intracellular (ICAM-1) and endothelial-leukocyte (E-selectin) adhesion molecules [45,46]. Moreover, HAECs supplemented with 1-3 mM ET significantly reduce binding by U937 human monocytes upon stimulation by IL-1β [46]. 

Previous studies have also demonstrated the anti-inflammatory properties of ET, including the earlier study in lung alveolar epithelial cells [37,38,45,131,132,133]. Collectively, these studies provide a strong case for ET supplementation to protect against endothelial dysfunction due to SARS-CoV-2 invasion of the endothelium and thereby mitigate associated pathologies such as cytokine storms, thrombosis formation, and cardiovascular events.

### 3.5. Effects on Hypercoagulative State and Ischemic Injury

Numerous reports have indicated an increase in thromboembolic events such as catheter line thrombosis, deep vein thrombosis and pulmonary embolism, ischemic stroke, and myocardial infarction in COVID-19 patients [134,135]. A study by Klok et al. [136] identified that out of 184 critically ill COVID-19 patients, about 31% suffered from thrombotic complications. The high levels of neutrophils and the promotion of NETosis mentioned earlier as part of COVID-19 pathology are known to play a key role in the promotion of vascular thrombosis [137]. In addition to binding pathogens, NETs have also been shown to bind to red blood cells, causing platelet adhesion and activation, leading to further aggregation, and eventually, a clot [138]. Studies have shown that the administration of DNases or the anticoagulant heparin can break up this NET aggregate before a thrombotic event can occur [138]. Indeed, the use of heparin in COVID-19 patients was associated with a decrease in severe outcomes and mortality [139], although other studies have found no benefits from thromboprophylaxis [135,140,141]. Some groups also highlight SARS-CoV-2 infiltration of the vascular endothelium, leading to inflammation and endothelial dysfunction (Section 3.4), as playing a role in thrombotic events in the microvasculature of COVID-19 patients [126,142]. Indeed, endothelial dysfunction is implicated in a wide range of cardiovascular disorders and promotion of the hypercoagulative state, which may lead to stroke, myocardial infarction, and acute kidney injury [127]. Some studies have further reported that COVID-19 patients are in a hypercoagulable state with elevated D-dimer and fibrin/fibrinogen degradation products, predisposing patients to thromboembolic events [135,140]. Although the exact mechanisms leading to the high risk of thromboembolism are not known, it is likely that a combination of the aforementioned factors could lead to the promotion of thrombotic events in COVID-19 patients. As mentioned earlier, ET may play a key role in the inhibition of NETosis, through the direct inhibition of MPO, inflammatory cytokines, and scavenging of ROS. ET may also play a role in the prevention of endothelial dysfunction, and both these actions suggest a role for ET in the prevention of coagulopathy in COVID-19.

A report from Italy has identified a number of cases of acro-ischemia (ischemia in digits), presenting as reddish-purple lesions, mostly in children or adolescents, and typically occurring in asymptomatic or mildly symptomatic COVID-19 patients [143]. Other reports related to acro-ischemic lesions have also surfaced, mostly in younger individuals [144,145]. Although not life-threatening (and not widely reported), these may be indicators of a more sinister underlying pathology, that is, the hypercoagulative state due to SARS-CoV-2 infection. A report in the *New England Journal of Medicine* described a number of cases of large vessel stroke in younger COVID-19 patients (below 50 years of age), with no underlying risk factors, and in some cases, before other symptoms of COVID-19 [146]. Additionally, a few reports from China have indicated that a higher incidence of myocardial injury and infarction was significantly associated with fatal outcomes in COVID-19 patients [147,148]. These reports all indicate the ability of the virus and/or resultant inflammation to cause damage to the vasculature, promoting a hypercoagulative state, and ischemic events to tissues and organs around the body. This, combined with the low oxygen levels due to respiratory injury, may contribute to multiorgan failure. It may also be pertinent post-COVID-19 since studies have shown that the risk of stroke and myocardial infarction are significantly elevated for many years following transient ischemic events in an individual [149].

Whether through thrombosis formation or respiratory deficiency, the hypoxic state can lead to organ and tissue injury. Furthermore, the restoration of oxygen to tissues (especially when a patient is placed on mechanical ventilation) can give rise to reperfusion injury [76,150]. A few earlier studies have demonstrated that ET is protective in tissues exposed to ischemia-reperfusion injury. Bedirli et al. [50] reported that preadministration of ET to rats protected the liver from ischemia-reperfusion injury and led to significantly increased 7-day survival rates. Likewise, Sakrak et al. [39] demonstrated that ET protected against intestinal ischemic injury in rats, revealing significantly lower levels of IL-1β, TNF-α, MPO, and lipid peroxidation, as well as improved tissue morphology in ET-treated animals relative to controls. Both studies also reported that ET promoted the expression of heat-shock protein-70 (HSP-70) [39,50], an endogenous chaperone protein known to be cytoprotective during tissue stress and low oxygen by modulating inflammation, protecting against ROS, and preventing apoptosis [151]. HSP-70 has also been shown to protect against ARDS and sepsis [152,153].

Studies have also suggested that ET is protective in preeclampsia, a hypertensive disorder during pregnancy that results in placental ischemia, by protecting against reperfusion-induced oxidative stress and inflammation, preserving mitochondrial function, and reducing hypertension in a reduced uterine perfusion pressure rat model of preeclampsia [65,66,154]. Conversely, mice deficient in OCTN1 (whose tissues are completely devoid of ET) are more susceptible to intestinal ischemia and reperfusion injury, with higher inflammatory markers and mortality compared with wild-type animals [101]. These studies reinforce earlier claims of the antioxidant, anti-inflammatory, and other cytoprotective functions of ET and further suggest that it may help protect against sepsis, ischemia, and multi-organ failure in COVID-19.

### 3.6. Protecting the Brain

There may be multiple mechanisms by which COVID-19 can lead to neurological symptoms, including respiratory distress leading to hypoxia, cytokine storm-induced inflammatory damage to the central nervous system (CNS), the hypercoagulable state leading to cerebral venous thrombosis or stroke, or by direct SARS-CoV-2 invasion of the brain. As with the earlier SARS-CoV [155,156], the presence of SARS-CoV-2 has been identified in the CNS of COVID-19 patients [157]. Evidence of neurological manifestations due to COVID-19 has been reported in about 36% of patients according to a study in China, with symptoms including headaches, dizziness, loss of consciousness, ataxia, acute cerebrovascular disease, seizures, neuralgia, and encephalopathy [157,158,159]. While mechanisms of viral entry have yet to be ultimately established, mounting evidence suggests that SARS-CoV-2 can access the CNS through peripheral or olfactory nerves [160,161,162], although this does not rule out entry through the vascular [163] or lymphatic systems [164]. Studies administering SARS-CoV or MERS-CoV intranasally to transgenic mice expressing human ACE2 or dipeptidyl peptidase 4 (DDP4) receptors (the receptor facilitating MERS-CoV infection), respectively, revealed that these coronaviruses could enter the brain apparently through the olfactory nerves, infiltrating neurons and spreading to other parts of the brain, predominantly the thalamus, cerebrum, and brainstem [162,165]. Analysis of transcriptomic databases shows that ACE2 is highly expressed in the substantia nigra, brain ventricles, middle temporal gyrus and posterior cingulate cortex, and various other regions of the brain, implying that the SARS-CoV-2 is indeed capable of infiltrating the brain, causing neurological symptoms [166]. Indeed, an increasing number of reports have indicated the loss of olfactory and gustatory functions in infected subjects [18,159]. The olfactory epithelium, located in the nasal cavity, also expresses the ACE2 receptor and is likely the primary site of infection by SARS-CoV-2. This suggests that the infection of these cells may be the underlying mechanism for loss of olfactory function and a means for viral entry into the CNS [161]. This is supported by the fact that around 86% of COVID-19 patients (in a European study of 417 patients) with mild–moderate severity reported olfactory dysfunction, of which 35% of cases reported anosmia before or at the same time as other common symptoms of COVID-19 [167]. Olfactory dysfunction is also an early preclinical indicator for Parkinson’s disease (PD) in greater than 95% of cases and may precede motor dysfunction by years [168,169,170]. In the case of PD, it is believed that damage to the nondopaminergic neurotransmitter systems (cholinergic, serotonergic, and noradrenergic systems) leads to olfactory loss, and also the induction of localized brain inflammation and oxidative damage due to dysregulation of the microglia [169,171]. While the mechanisms are likely to be different, the underlying damage to olfactory systems may also lead to the resultant induction of brain inflammation and oxidative stress. Certainly, the brain is more prone to elevated levels of oxidative stress [172,173], for example, in bacterial meningitis [174] or, relevant to viruses, HIV-induced neurocognitive disorders [175].

The pathological mechanisms underlying the neurological manifestations of COVID-19 are still not well understood. Autopsy reports from patients with COVID-19 in China identified the presence of neuronal degeneration and edema in the brain [159]. Li et al. [176] have suggested that the SARS-CoV-2 invasion of the brainstem, which regulates the cardiorespiratory function, may play a role in the pathophysiology of acute respiratory failure in some COVID-19 patients, although this hypothesis has yet to be validated. Indeed, further studies are critically needed to understand the mechanisms of infection and the impact and damage caused directly and indirectly (e.g., hypoxia, cytokine storm) by SARS-CoV-2 infection.

Additionally, the underlying damage to the CNS or disrupted homeostasis due to COVID-19 may lead to lasting neurological impairment even after the virus has been eradicated, which warrants further investigation into possible therapies to alleviate these impairments. Indeed, a viral infection of the CNS is suggested to be a risk factor for PD [177] (especially considering that olfactory damage is one of the early indicators of PD) and other neurodegenerative disorders such as Alzheimer disease, Down syndrome, and Lewy body dementia [169]. Indeed, an earlier study examined the CSF of PD patients and identified the presence of coronavirus antigens, suggesting a link between coronavirus infection and PD [178]. Hence, infection by SARS-CoV-2 and damage to the olfactory bulb may increase the risk of neurodegenerative disorders such as PD. In addition, some severe neurological events such as encephalopathy or cerebral ischemic stroke, as seen in about 20% of patients admitted into ICU, according to a study in France, may lead to permanent brain injury. Most neurodegenerative disorders [76,173,179,180] and stroke [76,181,182] are well known to involve oxidative stress; hence, this likely plays a role in the sustained damage.

ET has been shown to be protective against a range of neurotoxins in both in vitro and in vivo models, such as amyloid-β [58,59,183], cisplatin [184], D-galactose-induced dementia [185], and *N*-methyl-D-aspartate-induced cytotoxicity in rat retinal neurons [186]. Moreover, preclinical studies have revealed that ET preserved dopaminergic neurons and motor function in a 6-hydroxydopamine murine model of PD (manuscript in preparation). Studies have also shown that significantly lower blood levels of ET occur in patients with PD [74] and mild cognitive impairment [60] (an early stage of dementia) compared to age-matched controls, suggesting that lower levels of ET may be a risk factor for neurodegeneration. The mechanisms of protection by ET in these neurodegenerative models may involve protection against neuroinflammation and oxidative damage, prevention of mitochondrial dysfunction, metal chelation, or other neuroprotective mechanisms that have yet to be uncovered [59,61,184,185]. ET may be protective against SARS-CoV-2 infection of the brain by targeting these and perhaps other mechanisms. Furthermore, the prevention of a hypercoagulative state and protection of tissues against ischemia-reperfusion injury by ET, as mentioned earlier, may have implications in the prevention of or mitigation of damage during cerebral ischemic stroke that has been reported in a number of severe cases of COVID-19.

ET has been suggested to play a role in neuronal differentiation and maturation, which may be involved in repair following brain injury [51,52,53]. Studies have demonstrated that exposure of cultured hippocampal neurons to ET elevated the expression of the synapse formation marker synapsin-1 and neurotrophin-3 and -5, and ET supplementation in mice was shown to enhance learning and memory (object recognition test) and increase the number of mature spines in the hippocampus [51]. This ability may be useful following viral infiltration and damage to neurons in the brain.

Another important factor is that ET can cross the blood–brain barrier and accumulate in the brain following oral consumption, which is typically a major hurdle and a cause for the failure of many neuroprotective drug candidates, including antioxidant ones. Numerous reports have identified the presence of OCTN1 in the neurons from the hippocampus, hypothalamus, cerebellum, and motor cortex regions of the rodent brain [187]. As mentioned previously, ET is not rapidly cleared from the body like some other drugs and nutraceuticals and has high bioavailability to the brain, as witnessed from studies in animals [32,188,189] and postmortem human samples [24,190]. 

While the pathology underlying neurological symptoms in COVID-19 has yet to be fully uncovered, there is a strong likelihood that ET may play a protective role, especially when the pathology involves oxidative damage and inflammation. Certainly, further studies are needed and warranted to evaluate if ET may be neuroprotective against SARS-CoV-2 through these and other mechanisms.

### 3.7. Restoring Dysfunctional Iron Metabolism

The role of iron in the pathology of COVID-19 is still unclear. However, iron has been shown to play a role in the genome replication and protein translation by many viruses, and indeed chelating iron was demonstrated to restrict the propagation of viruses such as HIV, human cytomegalovirus, vaccinia virus, herpes simplex virus 1, and hepatitis B virus in vitro [191]. It is well-known that iron, ROS, and inflammation are interconnected, and excessive free iron generated through iron dysregulation or breakdown of iron-metalloproteins may exacerbate disease progression and severity through Fenton-generated ROS (that is the Fe^2+^-dependent production of ^•^OH from H_2_O_2_), in turn promoting further inflammation [76]. Disruption of iron homeostasis is known to be a contributing factor in many disorders of the brain, liver, kidneys, and, in particular, the lungs, including ARDS, whereby the excess free iron contributes to oxidative stress and further inflammation if left unchecked [76,90,192,193]. Indeed, elevated levels of total iron and non-haem iron were seen in the bronchoalveolar lavage fluid of ARDS patients compared to healthy controls [90]. Liu and Li [194] have even postulated that certain proteins in the SARS-CoV-2 virus may bind hemoglobin in red blood cells, dissociating the iron from haem, and causing damage whilst contributing to the impaired gas exchange in the lungs. However, there is no evidence as yet to support this hypothesis.

Elevated levels of ferritin (an iron storage protein) have been associated with cytokine storms and sepsis and linked with high mortality [195,196,197]. Indeed, hyperferritinemia has been observed in COVID-19 patients (even in asymptomatic cases) [198]. Some have suggested that this may be one of the underlying pathological mechanisms driving cytokine storms and sepsis in critically ill COVID-19 patients [199], and early reports have demonstrated that extremely high levels of ferritin were correlated to mortality [115]. Studies show that high ferritin levels are an indicator of macrophage activation (macrophage activation syndrome, which is seen in viral infections) [200] and ferritin itself might function as a proinflammatory cytokine [201] and hence may play a role in the propagation of a vicious cycle of inflammation, leading to a cytokine storm [197,202]. Free iron can be released from ferritin upon exposure to certain ROS [76]. This presents a case for the chelation of iron to reduce the severity of symptomology due to ARDS, cytokine storms, and sepsis, the three most common facilitators of mortality in COVID-19.

Indeed, earlier studies have demonstrated that ET is able to chelate divalent metal ions, including Fe^2+^ and Cu^2+^ [40,42,203], with high stability constants. This metal ion chelation forms redox-inactive complexes with ET, thereby preventing damage to biomolecules [33,41]. This would suggest that if free iron were to exacerbate the pathology of ARDS and complications in other organs associated with SARS-CoV-2 infection, ET may play a role in preventing this from promoting further oxidative stress and thus lessen damage to the lungs and other tissues and organs (especially the brain). As mentioned earlier, ET has been shown to modulate the expression of proinflammatory cytokines and thus may play a role in breaking the chain of hyperinflammation associated with macrophage activation and hyperferritinemia.

### 3.8. Protecting against Acute Kidney and Liver Injury

Initial reports from China have indicated that the prevalence of kidney injury due to COVID-19 is relatively low (between 0.5‒7% of hospitalized patients) [111]. However, more recent reports have suggested that the incidence is far higher than initially indicated, with one recent study of 5449 hospitalized COVID-19 patients in New York demonstrating that 1993 patients or 36.6% of the total patients admitted developed acute kidney injury (AKI), with 14% of these requiring dialysis [204]. AKI (associated with elevated serum creatinine and urea levels, as well as high levels of protein in urine) is typically associated with more severe symptoms, especially those requiring mechanical ventilation and is a prognostic factor for poor outcomes, with one analysis suggesting that incidence of AKI carried about 5.1 times the risk of mortality compared to patients without kidney dysfunction [205,206]. While studies have demonstrated that ACE2 is highly expressed in renal tubular [207] and epithelial cells [208], it is less clear whether AKI is due to direct viral invasion of the renal system or indirectly as a secondary casualty of impaired respiration, thrombotic events of the renal vein, or collateral damage resulting from the cytokine storm. However, evidence suggests the latter, as studies reveal a strong correlation between AKI and respiratory failure, with close to 90% of patients on mechanical ventilation showing manifestations of AKI [204]. This has also been observed in other studies, and furthermore, the first incidence of AKI closely follows the time of intubation and mechanical ventilation for most patients with respiratory failure, suggesting ischemia leading to necrosis of the renal tubular epithelium [204,209]. The elevation in IL-6 during a cytokine storm has also been suggested to be a contributing factor to AKI through damage of the renal tubular epithelium, which in turn further elevates IL-6, indicating bidirectional lung–kidney damage [210].

No specific treatments have been applied for AKI, with the exception of severe cases requiring renal replacement therapy, and COVID-19 therapies remain largely assistive for the management of ARDS and sepsis [205]. Indeed, ET would suit this therapeutic role. Studies in rodents have demonstrated that ET decreases oxidative damage in the kidneys and liver from the ROS-generating agent ferric-nitrilotriacetate [104]. By contrast, knocking out the ET transporter in murine models of chronic kidney disease increased kidney damage and fibrosis and elevated oxidative damage [105]. Moreover, blood levels of ET were found to be decreased in patients with chronic kidney disease, suggesting that low ET levels might play a role in the progression of renal injury and, conversely, that supplementation may be protective [105].

About one-third of patients admitted to hospitals also had abnormal liver function tests, and this was associated with a prolonged duration of disease [211]. Liver biopsies taken from COVID-19 patients indeed revealed the presence of liver damage, although the mechanisms are as yet unclear [112]. As with the kidney, the underlying mechanisms could be due to direct viral infection or as a result of immune-mediated damage or hypoxic hepatitis due to respiratory failure or drug-induced liver injury [212]. With the surge in repurposed drug trials and the development of novel therapies, the choice of treatment may also play a role in kidney or hepatic injury, e.g., remdesivir (a broad-spectrum antiviral) may cause liver damage and hence may not be suitable for COVID-19 patients with liver symptoms [213,214].

While hepatocytes do not typically express ACE2 receptors, some studies have suggested that the ACE2 expression may be upregulated following liver injury [215], while other groups have suggested that expression of ACE2 in the epithelial cells of the bile ducts may facilitate SARS-CoV-2 entry into the liver [216]. Regardless, numerous reports have indicated the elevation of liver enzymes, an indicator of liver damage [211]. Like the kidney, ET may also be beneficial in the liver, where earlier studies have demonstrated that it is the first site of rapid ET accumulation following oral administration in mice [32]. Additionally, we have shown that the liver can upregulate the ET transporter in response to damage, further increasing tissue ET levels, where it may be protective against oxidative damage, inflammation, and fibrosis formation [56,104]. Similarly, studies by Tang et al. [55] identified that human hepatic stellate cells upregulated OCTN1 following administration of dimethylnitrosamine (DMN), a hepatotoxin that increased levels of both oxidative damage and α-smooth muscle actin, the latter being a marker for activated stellate cells and liver fibrosis. Supplementation with ET decreased oxidative damage and DMN-induced activation of liver fibrosis, while conversely, knocking out OCTN1 resulted in a significant elevation of liver fibrosis markers, oxidative damage, and inflammation in mice [55].

It remains to be seen whether COVID-19 will result in any sustained renal or hepatic injury or whether lesions will promote fibrosis. However, it is becoming increasingly clear that reducing the severity of damage and duration of the illness may significantly decrease the chance of any long-term effects. Indeed, most patients with AKI who do not require dialysis usually recover after the clearance of the virus [217]. Moreover, care must be taken with the use of hepatotoxic drugs when liver damage is evident. Conversely, the administration of these drugs in combination with ET may be a viable approach. The evidence here suggests that the administration of ET may help protect the renal and hepatic tissues, prevent severe outcomes, and inhibit fibrosis formation, thereby lessening the chance of sustained damage due to COVID-19.

### 3.9. Protecting against Gastrointestinal Disorders

Instances of nausea, vomiting, and diarrhea have been reported due to SARS-CoV-2 infection and are typically associated with more severe outcomes [218]. Reports about the incidence of gastrointestinal manifestations are highly variable, with earlier reports indicating the frequency of between 1‒4% [15,111], while later studies found around 10% [16] and 60% [219] occurrence, suggesting that gastrointestinal symptoms may be often overlooked and are hence under-reported. Studies in both China and Singapore identified the presence of the virus in the gastrointestinal tract in about 50% of patients, and, furthermore, about 43% of these patients continued to test positive for the virus in stool samples even after the respiratory samples were clear of the virus [220,221]. The continued viral shedding in stool samples after the absence from nasopharyngeal swabs has been verified by other studies [222,223]. As with other tissues and organs implicated in COVID-19, ACE2 expression is abundant in the esophagus, gastric mucosa, small intestine, and colon [224] and immunofluorescent staining for viral nucleocapsid proteins of gastrointestinal epithelia, taken during endoscopic examination of a COVID-19 patient, demonstrated that SARS-CoV-2 can invade the epithelia of the stomach, duodenum, and rectum [221]. Although the underlying processes leading to manifestations of gut symptoms have yet to be uncovered, ACE2 is a vital regulator of intestinal inflammation [225], and hence viral binding is suggested to lead to intestinal inflammation and diarrhea [226]. In one patient, endoscopic examination revealed esophageal bleeding and ulceration, with SARS-CoV-2 RNA present at the site of injury [219]. While the underlying causes of gut manifestations, the prolonged presence of the virus in the gastrointestinal tract, and the possibility of long-term ramifications remain to be seen, thus far, the key instigator of gut symptoms appears to be inflammation.

As mentioned earlier, ET can limit inflammatory injury in the intestines of mice following ischemia-reperfusion, and this protection was removed by knockout of the ET transporter [101]. Additionally, lower serum levels of ET have been associated with Crohn’s disease, an inflammatory disorder of the intestines, and these lower ET levels have been suggested to exacerbate this inflammatory bowel disease (IBD) [227]. Studies have demonstrated that, indeed, high levels of ET are able to reach the various parts of the gastrointestinal tract and can be absorbed through the epithelia following oral administration [32]. Furthermore, a study of IBD in mice has demonstrated that OCTN1 expression and ET uptake were elevated in the gastrointestinal tract of the colitis model relative to controls [131], suggesting a possible adaptive response that can increase tissue ET levels following inflammation [57]. The authors also demonstrated that ET can enter the lamina propria mononuclear cells (LPMCs) isolated from the colitis murine model, but in OCTN1 knockout animals, ET uptake is absent and inflammation is aggravated [131], suggesting that ET uptake may prevent the activation of LPMCs and reduce inflammation in IBD [228]. These data suggest that ET may play a role in protecting the digestive system in COVID-19 patients.

### 3.10. Protecting against Gonadal Manifestations

Studies have commenced investigating the longstanding effects of COVID-19 infection on testicular function in males from Wuhan, China [229]. Bioinformatic studies of mRNA databases indicate that the testis has the highest expression of ACE2 receptors in the body, in particular, the Leydig cells and cells in the seminiferous ducts [207]. Earlier studies in SARS-CoV, which also targeted the same receptor, have revealed that numerous patients had evidence of testicular inflammation (orchitis) [230]. Indeed, a range of other viral infections such as HIV, mumps, hepatitis B, and human papillomavirus has been shown to cause male sterility, altered expression of hormones, and may even be linked to increased risk of testicular cancer [231,232,233,234,235]. One study demonstrated that the ratio of testosterone and follicle-stimulating hormone to serum luteinizing hormone was significantly decreased in young adult male COVID-19 patients (*n* = 81) compared to controls (*n* = 100) [229]. Numerous reports have demonstrated the presence of the ET transporter in the testis [101,236] and seminal vesicles [237] and that ET can accumulate in the testis and is abundant in testicular secretions [238,239,240]. Cisplatin, an antitumor drug, has been shown to cause testicular damage and Leydig cell dysfunction, resulting in infertility [241]. However, supplementation of rats with ET was shown to protect against cisplatin-induced reproductive toxicity, restoring sperm count, and decreasing oxidative damage and histological damage to the testis [242]. While the reason for the accumulation of ET in the testis is unclear, it is likely that its presence in these tissues will aid in protecting from orchitis or any other sustained injury due to COVID-19.

### 3.11. Restoring Decreases in the Elderly and Sick 

As mentioned previously, age and underlying comorbidities are highly associated with increasing disease severity and mortality due to COVID-19. There are multiple theories as to why advanced age stands out as the highest risk factor for mortality. With increasing age, there is a far higher chance of having underlying comorbidities and age-related disorders [243]. Moreover, with advanced age, there is also a decline in lung function [244], diminished immune function [245], and lower levels of certain endogenous antioxidant defenses, such as GSH [76,246] and increased underlying chronic inflammation [247]. These would all contribute to the impaired ability to neutralize the SARS-CoV-2 infection and exacerbation of critical symptoms, including ARDS, cytokine storms, and sepsis, amongst others. A recent study identified that SOD3 (encoding the extracellular Cu/Zn superoxide dismutase—an antioxidant enzyme that catalyzes the dismutation of O_2_^•−^ in the extracellular environment [76]) was the most downregulated gene in the alveolar type II cells of the lungs between elderly and young adults, and suggested this may be part of the reason for increased severity of COVID-19 in the elderly [248]. In addition, we and others have revealed that with advancing age, blood plasma levels of ET decline, and this may be a risk factor for age-related disorders and frailty [60,72]. Indeed, significantly lower plasma ET levels have been identified in a range of disorders relative to age-matched healthy controls [60,64,73,74]. Although the reasons for the decline are unclear, one could suppose that this may be due to depletion as a result of chronic disorders [246,249]. This would then provide the rationale that supplementation with ET may reduce the risk of age-related disorders or potentially act to counteract underlying pathologies such as oxidative stress and inflammation [24]. Indeed, a recent study by Smith et al. [64] found that higher plasma ET levels are an independent marker for lower risk of cardiometabolic diseases and mortality, while others have suggested that ET supplementation may promote healthy aging and longevity [71], and prevent frailty [72,73]. While the evidence presented previously suggests that ET may contribute in reducing the severity of symptoms and mortality, we propose that ET may also act as a prophylactic by reducing factors associated with aging and frailty and thereby reduce the chances of serious infection.

### 3.12. Protecting against Comorbidities

Numerous underlying comorbidities or factors have been associated with a high risk of complications and mortality due to COVID-19, including (but not limited to) diabetes, heart disease, hypertension, obesity, asthma, chronic obstructive pulmonary disease (COPD), PD, renal or hepatic disorders, and also individuals that smoke, are immunocompromised, or have certain genetic factors e.g., glucose-6-phosphate dehydrogenase (G6PD) deficiency [250,251,252,253,254,255]. For most of these underlying risk factors, the reason for the intensification of disease symptoms is likely due to the chronic inflammatory and imbalanced redox state in the body and diminished respiratory function in lung disorders such as asthma, COPD, pulmonary hypertension, or due to smoking. However, some other less obvious conditions may provide insights into the underlying pathologies and possibly lead to novel therapies for COVID-19.

One such example is individuals with G6PD deficiency, which has been suggested to cause vulnerability to coronavirus infection [254,255]. Reports have postulated that this is due to an imbalance of the endogenous antioxidant to the pro-oxidant pool in favor of the latter; hence, individuals with G6PD deficiency may not cope as well with the flux of ROS and inflammation [76,256]. Moreover, earlier studies have demonstrated that the elevated oxidative stress due to G6PD deficiency caused cells to be more vulnerable to coronavirus infection (increased uptake and viral replication) and that supplementation with antioxidants (such as α-lipoic acid) may help to protect G6PD-deficient individuals from viral infection [257]. Indeed, ET would be another likely candidate, having demonstrated not only the ability to act as an antioxidant but also to protect against the various other pathological mechanisms of COVID-19 described in this review, and may be of greater benefit to G6PD-deficient individuals during COVID-19. Another example is obesity. A study at one medical facility in France demonstrated that of the 124 COVID-19 patients admitted into ICU, about 48% had a BMI in the obese range, i.e., ≥30 (kg/m^2^), with about 90% of severely obese individuals requiring invasive mechanical ventilation [258]. A report by Kass et al. [259] revealed an inverse correlation between age and BMI in COVID-19 patients admitted to hospitals, that is, younger individuals with high BMI had more severe outcomes. Obesity is a major risk factor for many metabolic [260] and cardiovascular disorders [261], hence it is likely that many of these individuals may also suffer from one or more of the aforementioned comorbidities. Obesity itself is also a condition associated with chronic inflammation [262], elevated oxidative stress [263], and impaired immune function [264]. By protecting against this and other manifestations of COVID-19, ET may help reduce the severity and mortality of infection in these individuals. As mentioned earlier, studies found that higher blood ET was independently associated with a lower risk of cardiometabolic diseases that are commonly associated with obesity [64].

### 3.13. Protecting against Longstanding Effects Post-COVID-19 Infection

An alarming number of reports are beginning to expose the ability of the SARS-CoV-2 to spread through most areas of the body and cause damage to multiple tissues and organs [265]. Beyond the typical pneumonia-like symptoms of cough, fever and respiratory distress during infection of the respiratory system, reports have surfaced of other symptoms including ocular manifestations [266,267], testicular infection and injury [229,268], renal injury [269], diarrhea and other gastrointestinal problems, liver damage and cardiovascular complications, which poses the question whether there are any long-term clinical implications post-infection [111]. Evidence is now emerging that while the lungs are the primary target of the virus, the expression of ACE2 receptors in various tissues around the body allows the virus to infect many other regions of the body, including the brain, heart, blood vessels, kidneys, testicles, and gut [265]. This is especially due to the infiltration of the vascular and lymphatic systems [164]. The widespread distribution and accumulation of ET in tissues and vital organs throughout the body, even through oral administration, ensures a high and widespread bioavailability that could mitigate damage to these areas [32,101].

The ability of the SARS-CoV-2 to infect a wide range of tissues and organs poses the question of whether the damage caused will lead to any sustained injury or elevated risk of diseases long after the virus is cleared. Earlier studies in patients infected with SARS-CoV suggest that the duration and severity of illness are correlated with the likelihood of sustained injury post-infection [270]. Hence, therapeutics aimed at reducing the severity and duration of symptoms are of critical importance in preventing sustained injury. Many have emphasized the urgent need to gain a clearer understanding of the damage and develop drugs to facilitate the restoration of these organs [271]. Since the SARS-CoV-2 only emerged little more than a few months ago, little is known at present about whether the virus may elevate the risk of developing disorders in later life, such as cancer, neurodegenerative diseases, cardiovascular disorders, stroke, hypertension, and so on. Only time will tell; however, studies have shown that infections with certain other viruses can significantly increase the risk of disorders such as cancer and cardiovascular disease. For example, hepatitis B and C and human papillomavirus are linked to liver and cervical cancer [272], respectively, while cytomegalovirus infection has been associated with a significantly increased risk of cardiovascular disorders. Oxidative damage has been implicated in all of these viral-associated risk factors [76,273,274]. This, once again, highlights the potential benefit from the administration of ET.

Another long-standing effect may be pulmonary fibrosis. It arises from tissue remodeling, thickening of interstitial walls, and scarring of damaged lung tissue, which can lead to loss of respiratory function due to the hardening of the tissue between the alveoli. Prior studies in 2003, utilizing thin-section computed tomographic (CT) scans, revealed that about 62% of SARS patients had evidence of fibrosis, which was more prevalent in older patients and those with more severe disease symptoms [253]. A one-year follow-up of survivors of the SARS-CoV infection found that one-third of moderate-severity patients had persistent pulmonary impairment [275]. Another study found that 9 out of 11 patients had mild to moderate lung function damage even 7 years after SARS-CoV infection, with thin-section CT scans revealing interstitial thickening [276].

Initial reports from China revealed a “patchy shadow or ground-glass opacity” in CT scans of the lung present in all COVID-19 patients admitted, which some have suggested may be early indications of interstitial thickening leading to fibrosis [16]. Indeed, postmortem histological analyses of pulmonary tissues from COVID-19 patients revealed evidence of fibrosis, which was positively correlated to the duration of illness [270]. This suggests that a prolonged duration of lung injury and ARDS increases the chance of long-term scarring of the pulmonary tissues. Hence, reducing the severity and duration of respiratory illness is crucial in reducing long-term or even permanent lung damage.

Studies in interstitial lung diseases leading to pulmonary fibrosis strongly implicate redox imbalance in the pathogenesis and progression of these conditions. Certain redox agents, especially *N*-acetyl cysteine, have been suggested to attenuate fibroproliferative events [277,278]. ET may thus be a prime candidate in the prevention of pulmonary fibrosis, acting through multiple mechanisms. Indeed, studies have shown that ET not only protected lung epithelial cells from the damaging effects of cigarette smoke but also inhibited pulmonary epithelial-mesenchymal transition, a key event in pulmonary fibrosis, thereby suggesting that ET may prevent fibroid formation in the lungs [54].

## 4. Safety of Ergothioneine

ET is a naturally occurring compound present in many foods at low concentrations, with the exception of mushrooms, where it occurs at high levels [24,279]. Despite a large number of studies on ET in the literature, none has yet reported any toxicity due to its administration, even at high concentrations. Toxicology studies undertaken in rats reported no adverse effects after a single acute dose of 2000 mg/kg/day or a continuous dose of 725 mg/kg/day for 92 days, which would roughly equate to a dose of 124 (acute) or 45 g of ET/day in an average adult human [280]. Furthermore, no observable effects were seen on fertility, gestation, and delivery at 725 mg/kg/day, nor were any adverse clinical or physiological indications seen in the litter [280]. Schauss et al. [281,282] also established the absence of any mutagenic and clastogenic effects due to ET administration. US FDA approved the status of “generally recognized as safe” (GRAS), and additionally, the European Food Safety Authority has granted approval for the use of ET as a food supplement even in infants, toddlers, and pregnant and lactating women [283,284]. Our own studies have revealed no adverse clinical and psychological effects in healthy young adults (less than 30 years of age) following administration of 25 mg/day for 7 days [29]. Moreover, our ongoing clinical study in elderly subjects, given 25 mg/day thrice weekly for 1 year, has thus far observed no adverse clinical events (NCT03641404). To the best of our knowledge, there are no known contraindications with ET, and since every person possesses some ET in their bodies (the levels depending on their diet), the chances of this seem unlikely. Collectively, this demonstrates the safety of ET for clinical use and, together with evidence of its potential efficacy, provides a compelling case for the therapeutic application of ET in COVID-19 patients.

## 5. Concluding Remarks

The global effects of the COVID-19 pandemic are devastating, to say the least. The total number of infections and the death toll (despite a much lower rate of mortality) due to COVID-19 have far exceeded that of the earlier SARS and MERS combined. The ability of SARS-CoV-2 to invade a wide range of cells and tissues beyond the lungs gives rise to a broader range of symptomology, with extremes in the degree of severity ranging from asymptomatic to multi-organ failure. Without early indicators or consistent monitoring of patients, this will often mean that treatment of certain symptoms may occur too late. It is also of concern that the possibility of long-term damage to certain organs or the elevated risk of disorders in later life will potentially escalate the burden on healthcare systems.

Currently, treatments are mostly exploratory and ill-defined, especially considering the broad diversity of symptoms, populations, and underlying comorbidities. Despite huge efforts to develop vaccines and repurpose drugs, clinical evaluation takes time, and it may be many months to years before safe and viable options surface.

**Figure 1:**
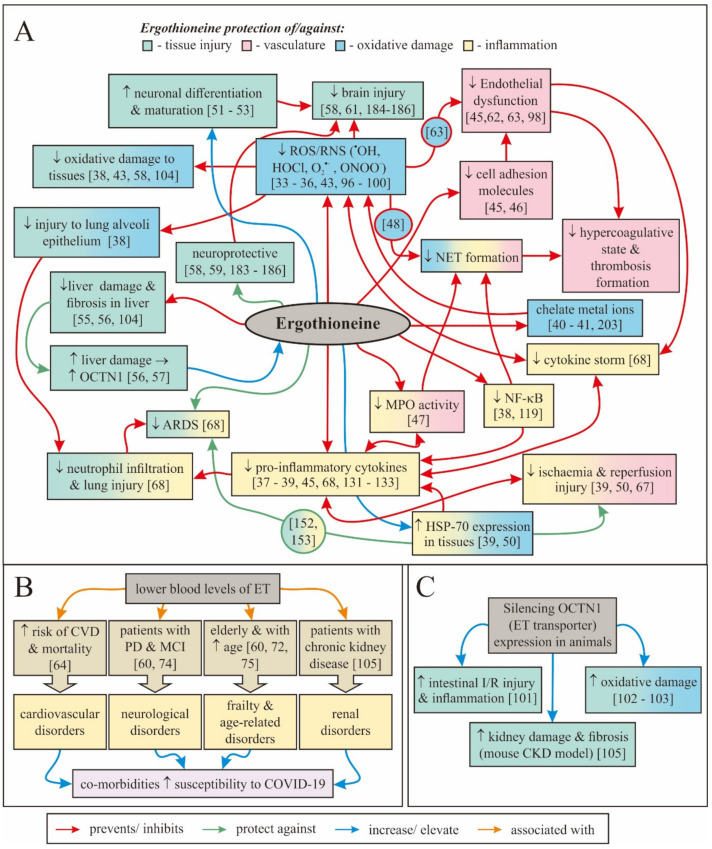
Summary of possible mechanisms of action of ET: (**A**) An overview of the possible direct and indirect mechanisms by which ET can reduce the severity of symptoms in COVID-19 patients and thereby reduce mortality [33,34,35,36,37,38,39,40,41,43,45,46,47,48,50,51,52,53,55,56,57,58,61,62,63,68,96,97,98,99,100,104,119,131,132,133,152,153,184,185,186,203]. (**B**) Population studies have shown that lower blood levels of ET are associated with a wide range of disorders and frailty, suggesting that supplementation may assist or reduce the risk of these conditions. These disorders are also comorbidities that likely increase the risk of mortality due to COVID-19, possibly highlighting the greater therapeutic value of ET for these individuals [60,64,72,74,75,105]. (**C**) Conversely, silencing the ET transporter in animal studies increases susceptibility to diseases and may elevate oxidative damage and inflammation in these models [101,102,103,105].

**Figure 2:**
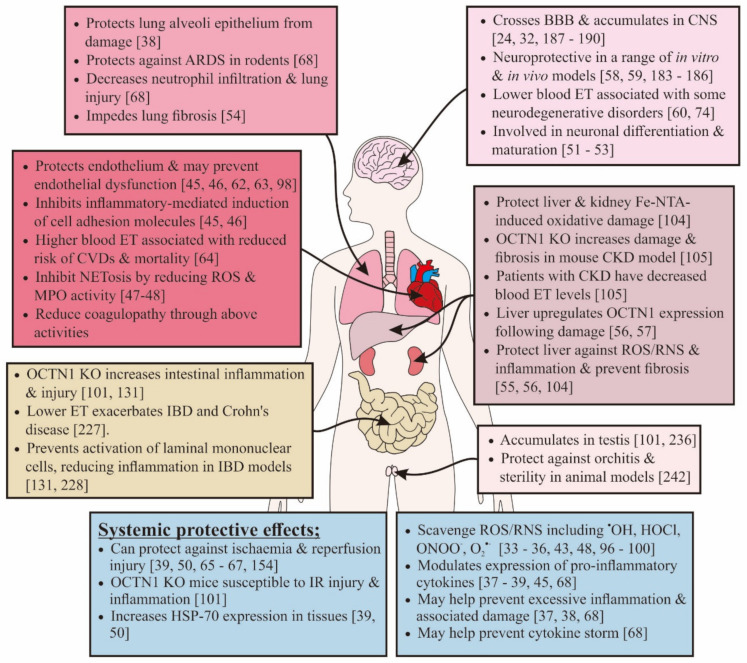
Summary of tissue protection by ET [24,32,38,45,46,47,48,51,52,53,54,55,56,57,58,59,60,62,63,64,68,74,98,101,104,105,131,183,184,185,186,187,188,189,190,227,228,236,242]. There is evidence to suggest that ET can accumulate in most (if not all tissues) in the body, especially those shown below. Based on present knowledge, the following diagram highlights how ET may protect various organs and tissues from oxidative damage and inflammatory injury amongst other cytoprotective effects in COVID-19 patients. The boxes in blue highlight systemic benefits for all tissues of the body [33,34,35,36,37,38,39,43,45,48,50,65,66,67,68,96,97,98,99,100,101,154].

This review presents the case for a safe, naturally occurring, and multifaceted compound, ET, as a therapeutic in reducing the severity and mortality associated with COVID-19 and improving prognostic outcomes of patients (Figure 1). A substantial number of studies on this unique compound have demonstrated its potential to reduce damage and underlying pathologies in a wide range of tissues (Figure 2). Indeed, ET has been shown to be readily absorbed and accumulated in most (if not all) tissues in the body. Although some evidence suggests that ET may play a direct role in mitigating infiltration and propagation of some viruses, studies need to be undertaken to establish if this is indeed the case for SARS-CoV-2. No studies have yet explored the application of ET for treatment and perhaps prophylaxis against COVID-19, and indeed, this is worthy of further investigation. 

Experts have speculated that complete eradication of SARS-CoV-2 will take far longer than anticipated, perhaps years, with pockets of resurgence in various communities around the world. Hence, there is an urgent need to test safe and novel treatments that may reduce the severity, duration, after-effects, and mortality associated with COVID-19.
